# Automated Discharge Instructions in Medical and Surgical Care: A Systematic Review of Patient Engagement and Clinical Outcomes

**DOI:** 10.3390/healthcare14060798

**Published:** 2026-03-20

**Authors:** Maissa Trabilsy, Ariana Genovese, Cesar A. Gomez-Cabello, Syed Ali Haider, Srinivasagam Prabha, Bernardo Collaco, Nadia G. Wood, Sanjay Bagaria, James London, Antonio Jorge Forte

**Affiliations:** 1 Division of Plastic Surgery, Mayo Clinic, Jacksonville, FL 32224, USA; 2 Department of Radiology AI IT, Mayo Clinic, Rochester, MN 55905, USA; 3 Department of Surgery, Mayo Clinic, Jacksonville, FL 32224, USA; 4 Center for Digital Health, Mayo Clinic, Rochester, MN 55905, USA; 5 Department of Artificial Intelligence and Informatics, Mayo Clinic, Jacksonville, FL 32224, USA

**Keywords:** health information systems, patient discharge, patient discharge summaries, computerized medical records systems, medical informatics applications

## Abstract

**Background:** Automated discharge instructions are increasingly used to support post-discharge communication, patient education, and nursing follow-up, yet the current state remains unidentified. This systematic review explores the types of automated discharge instructions used and their effectiveness in enhancing patient engagement and reducing readmission, emergency department visits and reoperation rates. **Methods:** A systematic search was conducted on 15 April 2025, using Embase, PubMed, Scopus, Web of Science, and CINAHL, following PRISMA guidelines. Inclusion criteria required peer-reviewed original research evaluating the utilization of automated patient discharge instructions following hospital admission or surgical stay. Exclusion criteria included correspondence, reviews, educational materials, not peer-reviewed, retracted reports, not retrievable, and no English translation. Risk of bias was assessed independently using NIH, JBI, ROB-2, and ROBINS-I tools. Two investigators independently conducted the screening, extraction, and synthesis of results using Endnote and Microsoft Excel. **Results:** Of the 1252 records identified, 13 studies were selected for analysis. There was a total of 34,386 patients across a diverse range of healthcare settings and clinical contexts. The average sample size per study was approximately 4912, with study samples ranging from 16 to 13,188 patients. The modalities of discharge instructions included automated phone calls (23.1%) and/or text messages (53.8%), as well as printed out auto-generated summaries (15.4%). Patient engagement was generally high, with automated phone calls showing the most consistent interaction, with completion rates ranging from 44% to 56%, often prompting clinical follow-up. SMS tools demonstrated strong scalability and response rates up to 87%. Two studies reported on hospital readmission outcomes and only a single study reported on emergency department revisit rates, while none assessed reoperation outcomes. Among those reporting readmission, automated phone calls and SMS were associated with lower or proxy-reduced readmission rates. Included studies had low to moderate levels of bias. **Conclusions:** While evidence on clinical outcomes such as readmissions, emergency department revisits, and reoperations remains limited and inconclusive, automated discharge tools—particularly phone calls and SMS—consistently demonstrated high patient engagement. Automated discharge tools show promise for supporting transitional care, discharge education, and post-discharge monitoring, highlighting the future role of automated tools in nursing workflows to support follow-up, escalation, and continuity of care.

## 1. Introduction

### 1.1. Background

Health literacy—the ability to obtain, process and understand basic health information and services needed to make appropriate health decisions—is a foundational component of effective healthcare delivery [1]. Yet, in the United States, the average patient reads at an approximate eighth-grade level, and many individuals lack the health literacy skills needed to navigate their medical care [2]. Recognizing this gap, the American Medical Association (AMA), National Institutes of Health (NIH) and Centers for Disease Control and Prevention (CDC) recommend that patient-facing materials be written at or below the sixth-grade level to accommodate the diverse literacy levels within the general population [2,3]. Despite these recommendations, patient discharge instructions frequently exceed this guideline by as many as four grade levels, creating an additional barrier to comprehension during a critical transition in care [2,4,5,6,7,8].

Even when written instructions are provided, their effectiveness is often limited. Printed discharge summaries are easily misplaced and may be misunderstood due to factors such as patient anxiety, cognitive impairment, or language barriers [9]. Not surprisingly, inadequate understanding of discharge materials has been associated with higher rates of hospital readmission, adverse events, and mortality [8,9,10]. These challenges highlight the need for more effective approaches to communicating essential post-discharge information.

In response, healthcare systems have explored alternative communication methods to enhance patient understanding at discharge. A growing body of research has evaluated the use of multimedia and technology-based tools as potential adjuncts to written or verbal patient education [11]. More recently, hospitals and ambulatory centers have begun implementing automated patient discharge tools—such as follow-up phone calls, text messages and auto-generated summaries—to support patients and families during the transition of care from hospital to home [12,13]. For nursing, these tools have the potential to extend discharge education beyond the point of hospital departure and support transitional care processes, including follow-up assessment, symptom monitoring, and reinforcement of care plans.

Automated discharge tools offer several advantages over traditional discharge communication methods. By leveraging technologies such as SMS, automated phone calls, and electronic summaries, these tools can deliver timely, standardized, and often personalized information directly to patients and caregivers [14,15]. Their scalability and ability to integrate with existing electronic health records make them efficient for wide deployment across healthcare systems [14,15]. Moreover, features like two-way messaging, reminders, and symptom check-ins have the potential to actively engage patients beyond the point of discharge, supporting better adherence, earlier identification of complications, and ultimately, improved outcomes [14,15].

As healthcare continues to embrace digital innovation, automated discharge tools represent a promising strategy to support nursing-led transitional care and continuity of care after hospitalization. However, the overall impact of these tools on patient engagement and clinical outcomes remains incompletely understood, particularly given their relatively recent adoption and heterogeneous implementation. Additionally, despite growing interest in automated discharge communication, this review is among the first to comprehensively assess their impact on patient engagement, hospital readmissions, emergency department revisits, and reoperation rates across medical and surgical populations.

### 1.2. Objective

The objective of this systematic review is to answer the following questions:

(1) What are the types of automated discharge instructions tools utilized in clinical settings?
(2) How effective are automated discharge instructions in enhancing patient engagement?
(3) How effective are automated discharge instructions in enhancing reducing hospital readmission, emergency department visits, and reoperation rates?

## 2. Methods

### 2.1. Search Strategy and Database Search

This systematic review was conducted in accordance with the Preferred Reporting Items for Systematic Reviews and Meta-Analyses (PRISMA) guidelines [16] and was registered in PROSPERO (ID: CRD420261321386). A search of five databases consisting of Excerpta Medica Database (EMBASE), PubMed, Scopus, Web of Science, and Cumulative Index to Nursing and Allied Health Literature (CINAHL), was conducted on 15 April 2025 with no time range filters. To optimize the retrieval of relevant articles, a search strategy was developed to use key terms related to automated patient discharge instructions. This review focused on studies evaluating the utilization of automated patient discharge instructions following hospital admission, emergency department visits or surgical procedures. A tailored search string for each database was created, using a combination of Medical Subject Headings (MeSH) and free text when applicable. Detailed queries can be found in the Supplementary Materials. All identified articles were imported into Endnote software (Version 20.4.1) for reference management.

### 2.2. Study Eligibility and Selection Process

The database search resulted in the identification of 1252 articles. Subsequently, 196 duplicate records were removed. Two independent researchers conducted the title and abstract screening and full text review based on the eligibility criteria, resulting in 13 studies included in the final analysis. A third reviewer resolved disagreements.

**Figure 1:**
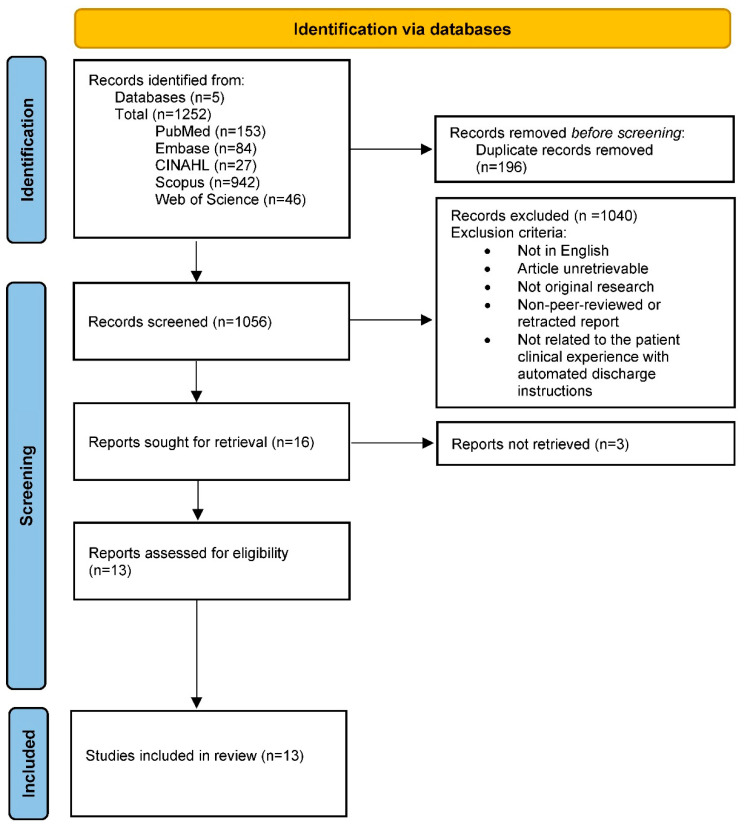
PRISMA flow diagram for study selection.

The inclusion criteria focused on studies which discussed the utilization of automated patient discharge instructions following hospital admission, an emergency department visit or surgery in peer-reviewed original research. Reasons for exclusion included unretrievable studies, correspondence, review articles, educational materials (book chapters), non-peer-reviewed or retracted reports, and lack of English translation. An overview of eligibility criteria can be found within the Supplementary Materials. Figure 1 demonstrates this process in accordance with the PRISMA guidelines.

### 2.3. Data Collection and Analysis

Study details and characteristics of each included study were systematically extracted and organized using Microsoft Excel by two researchers. The extracted details per article included the article type, year of publication and journal name. Details regarding the sample size, the type of automated patient discharge instructions used (via phone calls, text messages, or auto-generated printed summaries), whether the novel type of automated discharge instructions was more efficient than traditional discharge instructions pertaining to readmission rates, returning to the emergency room, returning to the operating room for re-operation, and/or general patient follow-up, and the main conclusions per study pertaining to patient engagement (the number of patients opening the discharge instructions, reading them and responding to them, if a response was warranted), and broader patient outcomes (adherence to medication and/or treatment plan, overall patient satisfaction and follow-up with healthcare providers). All articles which met inclusion criteria were extracted and no assumptions were made about missing data.

Due to the substantial heterogeneity in intervention types, patient populations, outcome measures, and reporting formats across the included studies, a quantitative meta-analysis was not feasible. Therefore, results were synthesized using a qualitative narrative approach to summarize patterns across interventions and outcomes. The data were summarized and synthesized via Microsoft Excel, BioRender and Microsoft Word to offer a comprehensive overview of what these studies depicted regarding the utilization of automated patient discharge instructions across medicine and surgery.

### 2.4. Risk of Bias Assessment

To assess bias in the selected papers, two researchers conducted an independent assessment using the National Institute of Health (NIH) Quality Assessment Tool for Before-After (Pre-Post) Studies with No Control Group [17] for four articles [18,19,20,21], the Joanna Briggs Institute (JBI) Critical Appraisal Checklist for Cohort Studies [22] for two studies [23,24], the Joanna Briggs Institute (JBI) Critical Appraisal Checklist for Cross-sectional Studies [25] for two studies [26,27], the Joanna Briggs Institute (JBI) Critical Appraisal Checklist for Case Series [28] for two studies [13,29], the Risk of Bias (ROB)-2 tool for Risk of Bias Assessment in Randomized trials [30] for two studies [31,32], and finally, the Risk of Bias in Non-Randomized Studies of Interventions (ROBINS-I) [33] for one study [34].

## 3. Results

### 3.1. Characteristics of Included Articles

The search conducted yielded a total of 1252 articles across five databases. Upon applying the inclusion and exclusion criteria, thirteen studies focusing on the evaluation of the utilization of automated patient discharge instructions following hospital admission, emergency department visits or surgical procedures were selected for this systematic review. All included articles were published from 2012 to 2025, with the majority published in 2025 [23,26,34]. A range of study types were included in this review, such as two randomized controlled trials [31,32], two descriptive observational studies [24,27], and three quasi-experimental (non-randomized) studies [19,20,21]. The included articles spanned twelve journals, with no single journal as the most common.

### 3.2. Risk of Bias

Table 1 shows a summary of the results of the included studies evaluated using the (NIH) Quality Assessment Tool for Before–After (Pre–Post) Studies with No Control Group [18,19,20,21]. The ROB-2 tool was used for Suffoletto et al., 2012 and Shuen et al., 2018, which were deemed as low risk and ultimately included in the review [31,32]. The ROBINS-I tool was used for Karasu et al., 2015, which was deemed moderate risk and ultimately included in the review [34]. The JBI Critical Appraisal Checklist for Cohort Studies was used for Bargas-Ochoa et al., 2025 and Olsen et al., 2016, which were both deemed low risk and ultimately included in the review [23,24]. The JBI Critical Appraisal Checklist for Cross-sectional Studies was used for Lu et al., 2025 and Ojeda & Kara 2017, which were both deemed as moderate risk and ultimately included in the review [26,27]. Finally, the JBI Critical Appraisal Checklist for Case-Series was used for Rozanec et al., 2017 and Wright et al., 2018, which were both deemed as low to moderate risk, and ultimately included in this review [13,29]. Overall, the methodological quality of the included studies ranged from low to moderate risk of bias across assessment tools.

### 3.3. Characteristics of Included Patients

The thirteen studies included collectively represent a total of 34,386 patients across a diverse range of healthcare settings and clinical contexts. The average sample size per study was approximately 4912, with individual study sizes ranging from 16 to 13,188 patients. Populations included adult and pediatric patients discharged from emergency departments, trauma and surgical units, radiation oncology, and general inpatient services. Several studies focused on specific groups, such as cancer surgery patients, tobacco users, or individuals with elevated blood pressure, while others evaluated broader cohorts with varied conditions. Table 2 describes a summary of the studies included in this review.

### 3.4. Characteristics of the Automated Discharge Instructions

Across this review, a variety of automated discharge instruction tools were utilized, reflecting the growing diversification of digital health strategies aimed at improving post- discharge care. The most common format was SMS (short message service or text messages), used in seven studies (53.8%) [18,20,21,26,31,32,34]. These interventions ranged from unidirectional pre- and post-operative reminders to bidirectional systems capable of receiving patient responses and prompting follow-up actions. Automated phone calls were employed in three studies (23.1%) [24,27,29], where they were typically utilized to assess post-discharge status within 24–72 h and connect patients to live support when needed.

**Figure 2:**
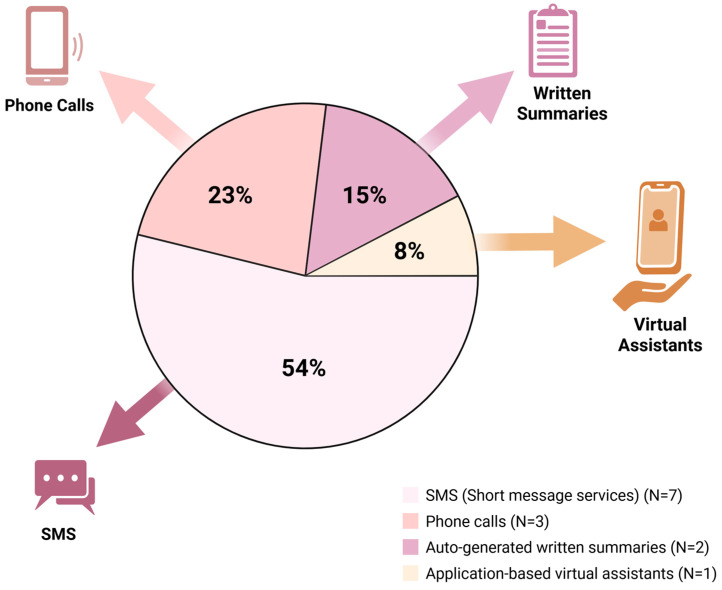
Distribution of automated discharge instructions across included studies. Created in BioRender [35].

Auto-generated written discharge summaries were utilized in two studies (15.4%) [13,19], providing patients with structured summaries at discharge. A single study (7.7%), Bargas-Ochoa et al., 2025 [23], implemented an app-based virtual assistant, delivering interactive, voice-enabled recovery instructions and symptom tracking via a tablet interface. Collectively, these tools ranged from simple reminder systems to sophisticated digital platforms, highlighting the breadth of strategies being evaluated to enhance patient understanding and continuity of care after hospital discharge. Figure 2 depicts the most used modalities of automated discharge instructions across the included studies.

### 3.5. Patient Engagement with Automated Discharge Instructions

Patient engagement with automated discharge instructions varied by tool type across the thirteen studies included in this review but overall demonstrated moderate to high responsiveness, acceptability, and satisfaction. Of the 34,386 patients represented across all studies and automation types, at least 33,129 (96%) reported measures of engagement such as message response, instruction access, or patient-reported feedback. One study demonstrated that out of 6867 patients contacted via automated phone calls, 3035 patients (44%) completed all five assessment questions by pressing keys on their phone that corresponded to their answers [24]. Another study that included 7246 patients reported that the global response rate to SMS was 87%, with 6343 patients responding to at least one SMS message [20]. Additionally, a study focused on medication adherence demonstrated that 67% of the 144 patients included responded to the SMS messages about their antibiotic prescription [32]. Another study showed that out of 251 total patients, twenty-eight subjects (34%) in the text group and 57 subjects (55%) in the phone group were successfully contacted at 48 h post-discharge [31]. The study utilizing a personal virtual assistant tablet app demonstrated that all sixteen surgical oncology patients completed daily check-ins, recovery tasks and symptom logs. Interestingly, another study that included 618 participants found that patients with higher education levels were significantly more likely to understand and report confidence in SMS-based instructions.

Five studies [13,23,26,31,34] reported subjective measures of engagement, three of which utilized validated or structured scales [23,26,34], with the remaining two studies [13,31] using informal or non-validated surveys post-discharge. One study utilized the SF-36 Quality of Life scale to assess emotional functioning and satisfaction as well as physical symptoms post-discharge for patients receiving discharge information via SMS [34], while another used the Technology Acceptance Model (TAM)-based survey, which assessed ease of use, usefulness, and patient attitude using a five-point Likert scale for a personal virtual assistant [23]. The third study utilized a questionnaire that was designed by the research team to evaluate domains of knowledge, care confidence, anxiety and satisfaction for SMS-based health discharge instructions, and also underwent a rigorous review for validation [26]. The remaining two studies [13,31] developed custom, non-validated patient satisfaction surveys to assess satisfaction with care, follow-up adherence and communication over the phone. Rozanec et al., 2017 also reported that only 45% surveyed received the eScribe-autogenerated discharge summary via physical handoff [13].

Overall, automated tools that included interactive features, personalization, or follow-up reminders tended to drive higher engagement, while one-way notifications showed more variable results. Though not all studies reported exact message open or read rates, the available data suggest that digitally delivered discharge instructions, particularly those with two-way communication capabilities, can effectively engage diverse patient populations in their recovery and transition of care. It is interesting to note that while SMS tools reached a larger volume of patients, and showed good engagement, automated phone calls had the highest consistent rate across diverse patient populations [24,29,31], especially when accounting for the depth of interaction, like the completion of assessments and follow-up care.

### 3.6. The Effect on Revisit to the Emergency Room Rates

Among the included studies in this review, only a subset directly evaluated the effect of automated discharge instructions on emergency department (ED) revisit rates. Only a single study reported on this outcome: Shuen et al., 2018, a randomized controlled trial of 251 patients discharged from the ED [31]. This study compared usual discharge instructions to follow-up via phone call or SMS. Although results did not reach statistical significance, the phone and text groups demonstrated over a 50% relative reduction in ED revisits compared to the control group [31].

In contrast, Chiu et al., 2022 investigated the effect of an automated discharge module on 30-day follow-up for ED patients with elevated blood pressure but did not assess ED return visits as an outcome, focusing instead on outpatient primary care follow-up [19]. Similarly, other studies, Lu et al., 2025 and Suffoletto et al., 2012, focused on patient understanding, medication adherence, or satisfaction, but did not report data on return visits to the ED [26,32].

### 3.7. The Effect on Readmission and Reoperation Rates

Few studies discussed the effect of automated discharge instructions on hospital readmission rates. The most detailed and statistically robust data set is described in Olsen et al., 2016, which included 6867 discharged patients contacted via an automated phone assessment within 24–72 h [24]. Among those who answered all five questions, the 30-day readmission rate was 5.0%, compared to 8.4% for patients who did not respond to the assessment. Notably, patients that were out of reach were almost two times more likely to have a readmission than those who answered all five assessment questions [24].

Peuchot et al., 2020 evaluated 4388 patients undergoing outpatient surgery. The use of SMS before and after surgery was associated with a conversion to full-time hospitalization rate of 0.36%, significantly lower than the 1.2% in the telephone group [21]. While technically this refers to unplanned admissions, it serves as a proxy for safety and could reflect reductions in unanticipated readmissions. Two other studies [20,27] monitored post-operative symptoms and issues through SMS and call-back programs but did not quantify readmission rates, although they discussed symptom detection and patient triage to potentially prevent readmissions.

Another study implemented an auto-generated discharge summary in radiotherapy patients but did not measure readmissions directly [13]. This study reported patients using their respective summaries in subsequent medical encounters, suggesting potential downstream benefits for continuity of care [13]. Similarly, Wright et al., 2018 suggested that post-discharge phone calls may be associated with reduced readmissions, but did not provide quantifiable data on readmission rates [29]. Instead, this study emphasized qualitative insights and laid the groundwork for future longitudinal tracking to assess this outcome more formally.

Notably, no study in this review reported surgical reintervention or reoperation rates, a critical outcome in post-surgical populations. Despite the fact that several studies involving surgical patients were included in this review, none assessed whether the use of automated discharge tools influenced the incidence of post-operative complications requiring surgical re-intervention. This absence highlights a critical evidence gap, particularly in surgical populations, where reoperation is a meaningful quality and safety indicator.

Table 3 summarizes the directionality of effects (positive, negative, neutral or not reported) across included studies, grouped by reported outcomes such as readmission, ED revisit, and patient engagement.

## 4. Discussion

### 4.1. Key Findings

This systematic review highlights several key findings regarding the use and impact of automated discharge instructions across medical and surgical care.

#### 4.1.1. Most Used Type of Automated Discharge Instructions

SMS (short message service) emerged as the most frequently implemented modality, utilized in over half of the studies (seven out of thirteen; 53.8%). These tools were leveraged for a range of functions, from one-way reminders to bidirectional messaging systems for symptom monitoring and patient feedback.

#### 4.1.2. Type with Highest Patient Engagement

While SMS platforms reached the largest volume of patients and demonstrated high engagement (response rates up to 87%), automated phone calls yielded the most consistent depth of interaction. Completion rates for phone call assessments ranged from 44% to 56%, and they were particularly effective in prompting follow-up care, suggesting that while SMS provides broader reach, phone calls may foster richer engagement.

#### 4.1.3. Association with Lower Readmission, ED Revisitation, and Reoperation Rates

**Readmission Rate:** The most robust data came from an automated phone call intervention, where patients who completed all assessment questions had a significantly lower 30-day readmission rate (5.0%) compared to those who did not respond (8.4%). SMS interventions also suggested lower proxy-readmission rates, particularly in surgical contexts where the conversion to full-time hospitalization was reduced.

**ED Revisitation Rate:** Only one study directly evaluated ED revisit rates, showing a >50% relative reduction in revisits among patients who received follow-up via SMS or phone call, although not statistically significant.

**Reoperation Rate:** No study in the review reported data on surgical reintervention or reoperation, marking a notable gap in literature and a prime area for future investigation.

These findings underscore the growing utility of automated discharge instructions, particularly SMS and phone-based systems, in improving post-discharge communication and potentially reducing adverse outcomes. However, the limited and variable outcome data, especially regarding ED revisits and reoperations, highlight the need for more targeted, outcome-driven research. While high engagement and satisfaction rates position automated discharge instructions as promising tools, their true clinical utility remains uncertain without consistent data on downstream health outcomes. The limited reporting on readmission and revisit rates, as well as the complete absence of reoperation outcomes, indicates that many current implementations prioritize feasibility and user experience over rigorous outcome validation. Robust evaluation of clinical endpoints will require larger cohorts, standardized definitions, and improved integration with health record systems to ensure meaningful and generalizable findings.

### 4.2. The Current Landscape

The current landscape of automated discharge instructions reflects a healthcare system in transition, increasingly adopting digital tools to enhance post-discharge communication. For nursing, this transition is not merely technological; it represents a potential reconfiguration of post-discharge accountability—shifting discharge education and surveillance from a time-limited event at bedside to a longitudinal process that requires ownership, response capacity, and documentation.

This review highlights the growing implementation of automated discharge instructions, particularly via SMS and automated phone calls, across varied clinical settings including emergency departments, surgical units, and oncology care [13,19,24,31]. These tools range from basic reminder systems to advanced, bidirectional platforms offering symptom monitoring and real-time triage support [19,20,24,29]. Although these technologies differ substantially in complexity and design, ranging from simple SMS reminders to more sophisticated digital platforms, they share the common feature of automating the delivery or reinforcement of discharge information without requiring real-time clinician initiation. For the purposes of this review, they were grouped under the broader category of automated discharge instructions to reflect this shared functional goal. This diversification mirrors broader trends in digital health innovation, where scalability and personalization are becoming critical to patient-centered care delivery. However, scalability in communication can unintentionally create scalability in demand for clinical response—particularly for nurses—unless the intervention is paired with explicit operational pathways.

Patient satisfaction with automated discharge instructions has consistently been high. SMS platforms have demonstrated engagement rates up to 87% [20], while automated phone assessments had completion rates between 44% and 56% across diverse populations [24,29,31]. Studies using validated measures, such as the Technology Acceptance Model (TAM) and custom satisfaction instruments, showed that patients found these tools intuitive and helpful, often reporting improved understanding and adherence to care instructions [23,26,34]. These findings are consistent with broader evidence suggesting that digital health tools, when appropriately designed, can enhance patient empowerment and confidence during care transitions.

Critically, empowerment is clinically meaningful only when the health system is prepared to respond. For nursing teams, higher engagement may increase the volume of patient questions, symptom reports, and uncertainty that would otherwise remain unexpressed, creating both an opportunity for earlier intervention and a risk of unmanaged workload if response systems are not defined. As such, Li et al., 2022 found that personalized mobile communication tools significantly increased medication adherence and reduced post-discharge anxiety in cardiac patients [36], highlighting the potential for such tools to complement nursing follow-up by reinforcing care plans and addressing patient concerns beyond discharge. However, high patient engagement does not necessarily correlate to improved clinical outcomes, as engagement metrics such as response rates or message interaction primarily reflect patient participation rather than direct measures of clinical benefit. Engagement should therefore be interpreted as a process indicator reflecting successful communication rather than a definitive marker of improved health outcomes. Furthermore, patient satisfaction with these tools has the potential to evolve and improve, with the integration of artificial intelligence in the near future, providing patients with a comprehensive resource regarding their surgical or medical care, its risks, benefits and possible outcomes [37,38].

Institutionally, however, the integration of automated discharge instructions into clinical workflows remains inconsistent. While some hospitals have incorporated the utilization of automated discharge instructions into electronic health record (EHR) systems or paired them with nurse-led care coordination programs [19,24], others encounter implementation barriers such as limited IT infrastructure, competing priorities, or insufficient staff training [39,40]. Additionally, differences in healthcare systems internationally may influence the implementation and effectiveness of automated discharge tools. Healthcare delivery models vary widely in terms of care coordination structures, reimbursement models, digital infrastructure, and nursing responsibilities in transitional care. As a result, the generalizability of findings across healthcare systems should be interpreted cautiously.

This mirrors findings from broader implementation literature, which emphasize that successful adoption of digital health interventions depends not only on the tool’s effectiveness but also on contextual factors like leadership support, organizational readiness, and user engagement [41,42]. Greenhalgh et al., 2004 and Cresswell et al., 2013 have both highlighted that the diffusion of innovations in healthcare relies heavily on local context and adaptability to clinician workflows [41,42]. This adaptability must be specified operationally: for example, who is responsible for monitoring incoming responses, expected response times, and how actions are documented and communicated to the broader care team. Without these elements, implementations may appear equivalent technologically while functioning very differently clinically.

Moreover, the lack of standardized protocols for the deployment of automated discharge instructions leads to variability in design, content, and frequency, making it difficult to establish best practices or compare effectiveness across settings [13,27]. While some systems utilize unidirectional reminders, others implement comprehensive platforms with interactive check-ins and follow-up prompts. This heterogeneity presents challenges for scalability and rigorous evaluation. In this review, another source of heterogeneity arose from the diversity of patient populations included, and the discharge needs and follow-up requirements of groups differ substantially. These differences may influence both engagement patterns and the potential clinical impact of automated discharge interventions. In nursing, heterogeneity also obscures key safety and quality dimensions: two interventions may both be labeled “SMS follow-up,” yet differ in whether they generate actionable symptom reports, whether messages are monitored by clinicians, whether escalation pathways exist, and whether patients receive clear guidance on what to do if symptoms worsen. These are not peripheral implementation details; they are care-process determinants that shape risk, workload, and the likelihood of meaningful clinical benefit.

Against this backdrop of variable design and implementation, automated discharge instructions continue to gain traction as a promising strategy to improve transitional care. Their favorable reception among patients, combined with growing institutional experimentation, suggests that automated discharge instructions have the potential to become a standard component of discharge planning. However, to fully realize this potential, future efforts must focus on standardization, provider engagement, and institutional capacity-building. Aligning these tools with broader health system goals and infrastructure will be essential for sustainable, widespread adoption.

**Figure 3:**
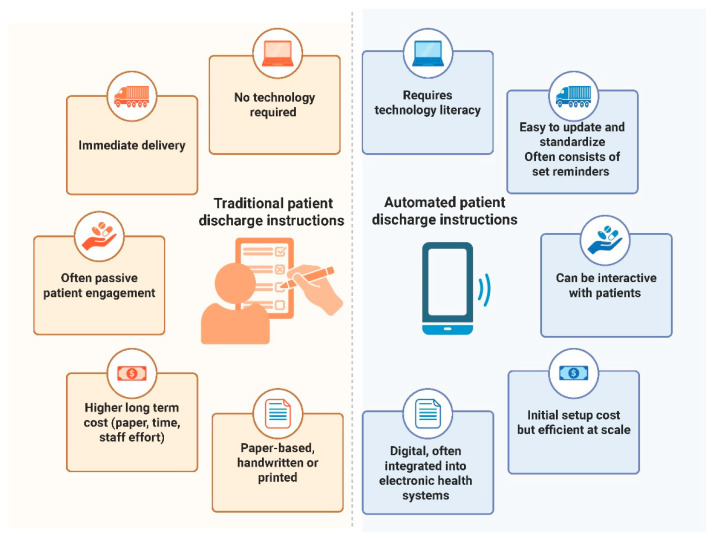
Subjective measures of patient engagement reported across included studies. Created in BioRender [43].

While automated discharge instructions differ significantly from traditional paper-based methods in format, delivery, and scalability, there is currently no definitive evidence suggesting that one approach is clinically superior. Automated tools offer advantages such as real-time delivery, scalability, and personalization, whereas traditional instructions remain accessible and familiar, particularly in low-resource settings. Both formats have unique strengths and limitations, and no form has yet demonstrated conclusively better outcomes in terms of readmission, ED revisit, or reoperation rates. As such, future research should focus not on format alone but on which specific features, such as clarity, interactivity, or follow-up support, best support patient understanding and recovery. This contrast is visually summarized in Figure 3, which outlines the key differences in format, delivery, and engagement potential, as well as the resource requirements between the two information dissemination methods.

### 4.3. The Limitations and Strengths of Review

This systematic review has several limitations that warrant consideration. First, there was a notable lack of studies reporting directly on key clinical outcomes such as hospital readmission, emergency department (ED) revisits, and reoperation rates. Only two studies evaluated readmissions, one assessed ED revisits, and none reported reoperation data, limiting the ability to make definitive conclusions regarding the clinical impact of automated discharge instructions. Second, the included studies exhibited substantial heterogeneity in design, patient populations, care settings, and intervention formats. This variability prevented formal meta-analysis and complicated cross-study comparisons. Third, measures of patient engagement were inconsistently defined and assessed. While some studies employed validated tools or structured surveys, others relied on informal or non-validated methods, which limits the comparability and generalizability of findings. Furthermore, given the relatively recent adoption of automated discharge instructions in clinical practice, most studies reflect early stage implementation, and long-term outcome data remain sparse.

Another important consideration relates to the influence of smaller or underpowered studies on the overall interpretation of findings, reflecting the emerging nature of the field. Several included studies had limited sample sizes or were pilot implementations evaluating feasibility rather than clinical outcomes. Although these studies contribute valuable insights into usability and early adoption, their statistical power to detect outcome differences is limited. If considered in isolation, such studies may over- or under-estimate the apparent effectiveness of automated discharge interventions.

Despite these limitations, this review possesses several important strengths. The review spans a diverse array of clinical disciplines, including emergency medicine, surgery, oncology, and general inpatient care, enhancing the relevance of its findings across healthcare contexts. Unlike prior literature focusing narrowly on usability or satisfaction, this study integrated both patient engagement metrics and clinical effectiveness outcomes, offering a more holistic assessment of the impact of automated discharge instructions. Furthermore, the review provides valuable insight into the current state of the evidence and clearly delineates existing knowledge gaps, particularly regarding surgical reintervention outcomes, thereby informing future research priorities in digital discharge communication.

In addition to methodological gaps, equity remains a critical but underexplored dimension in the implementation of automated discharge tools. Lu et al., 2025 observed that patients with higher education levels were significantly more likely to understand and feel confident using SMS-based instructions, pointing to disparities in digital health literacy [26]. However, few studies considered other key equity factors such as language barriers, age, socioeconomic status, and access to mobile technology. Veinot et al., 2018 has shown that limited digital access and literacy can significantly hinder engagement with health technologies in underserved populations [44]. Moreover, most implementations of the automated discharge instructions in this review occurred in high-resource, English-speaking environments, limiting generalizability. As Crawford & Serhal 2020 argue, equity-centered design is essential to prevent digital tools from reinforcing existing disparities [45]. Future research should adopt equity frameworks, stratify outcomes by social determinants, and develop culturally and linguistically inclusive automated discharge instructions to ensure broader and fairer impact.

### 4.4. Future Research

Future research should aim to address the critical gaps identified in this review, particularly by generating more robust and consistent data on the clinical effectiveness of automated discharge instructions. One of the most pressing needs is for high-quality studies that directly evaluate the impact of automated discharge instructions on objective health outcomes such as hospital readmission, emergency department (ED) revisits, and especially, surgical reoperations, an outcome notably absent from the current literature. To evaluate between various populations, subgroup analyses should also be conducted. Given the importance of reoperation as a quality and safety indicator in surgical populations, future studies in surgical settings should specifically track post-discharge complications and the need for reintervention. To meaningfully interpret these outcomes, studies should also describe how automated discharge instructions are embedded within discharge and post-discharge workflows, including who is responsible for monitoring patient responses and initiating follow-up or escalation. Without this context, it remains difficult to determine whether null findings reflect limitations of the technology or gaps in care-process integration.

Additionally, there is a need for standardized measures of patient engagement. Many of the included studies used informal or inconsistent engagement metrics, which limits comparability and reproducibility. Future studies should adopt validated engagement and satisfaction instruments, and clearly report metrics such as message open rates, response rates, and adherence to follow-up instructions. Studies should also investigate whether increased engagement in this context leads to earlier detection of complications, improved adherence to care plans, or enhanced coordination with healthcare teams, which may ultimately influence clinical outcomes.

Longitudinal research is also needed to assess the sustained impact of automated discharge instructions beyond the immediate post-discharge period. Most current evidence is limited to short-term outcomes, and it remains unclear whether benefits such as reduced readmission or improved engagement persist over time. Randomized controlled trials (RCTs) with longer follow-up periods could help determine the durability of ADI effects on patient outcomes and healthcare utilization.

Moreover, future research should explore the differential effectiveness of various automated discharge instruction modalities, such as SMS, automated phone calls, app-based tools, and written summaries, across diverse patient populations, including those with limited health literacy, non-English speakers, and those from socioeconomically disadvantaged backgrounds. Understanding how factors such as education level, age, language, and digital literacy influence the effectiveness of automated discharge instructions will be critical to ensuring equitable access and optimizing implementation. Studies should also assess how nursing involvement moderates the effectiveness of different modalities, particularly for patients who do not readily engage with digital tools or who require alternative follow-up strategies. Emerging agentic AI systems, which are capable of adapting content and delivery based on individual patient needs and context, may offer scalable, personalized solutions to these challenges. Their ability to dynamically tailor interactions could enhance engagement and comprehension across diverse populations.

Beyond the communication modality itself, future studies should also evaluate the content and readability of automated discharge messages. While this review categorized interventions primarily by technology type (e.g., SMS, phone calls, app-based tools), the informational clarity, tone, and readability of text-based messages may significantly influence patient comprehension and engagement. Incorporating standardized readability metrics may help ensure that automated discharge instructions remain accessible to patients with varying health literacy levels.

Advances in natural language processing and large language models (LLMs) may also offer new opportunities to systematically evaluate and optimize discharge communication. More broadly, emerging research in human–machine communication systems highlights how advanced computational models are increasingly being used to interpret and facilitate interactions between humans and digital technologies, suggesting potential future applications for personalized healthcare communication systems [46]. However, the implementation must be approached cautiously, as the use of patient-generated data raises important considerations regarding privacy, data security, and regulatory compliance (e.g., HIPAA). Future work should therefore explore secure, privacy-preserving approaches to applying these technologies within clinical communication systems.

Finally, economic evaluations should be integrated into future trials to assess the cost-effectiveness of automated discharge instruction programs. While these tools show promise in improving patient engagement, their scalability and integration into clinical workflows must be justified by demonstrated value in reducing complications and lowering healthcare costs.

## 5. Conclusions

This systematic review depicted high patient engagement, underscoring the potential for broader use in medical and surgical patient care. However, the results were inconclusive regarding the effect of automated discharge instructions (via phone calls, text messages and/or auto-generated printed summaries) on revisits to the emergency department, as well as readmission and reoperation rates. These findings suggest that automated discharge instructions can be effective at engaging patients but that their clinical impact likely depends on integration into discharge and transitional care workflows, particularly nursing-led follow-up, monitoring, and escalation.

## Figures and Tables

**Table 1:** Risk of bias assessment for selected studies using the NIH Quality Assessment tool; criterion defined as satisfied (✔), not reported (NR), not applicable (NA), or not satisfied (X).

Criteria	Chiu et al., 2021 [18]	Chiu et al., 2022 [19]	Leconte et al., 2019 [20]	Peuchot et al., 2020 [21]
1. Was the study question or objective clearly stated?	✔	✔	✔	✔
2. Were eligibility/selection criteria for the study population prespecified and clearly described?	✔	✔	✔	✔
3. Were the participants in the study representative of those who would be eligible for the test/service/intervention in the general or clinical population of interest?	✔	✔	✔	✔
4. Were all eligible participants that met the prespecified entry criteria enrolled?	NR	NR	✔	✔
5. Was the sample size sufficiently large to provide confidence in the findings?	X	X	X	X
6. Was the test/service/intervention clearly described and delivered consistently across the study population?	✔	✔	✔	✔
7. Were the outcome measures prespecified, clearly defined, valid, reliable, and assessed consistently across all study participants?	✔	✔	✔	✔
8. Were the people assessing the outcomes blinded to the participants’ exposures/interventions?	NA	NA	NA	NA
9. Was the loss to follow-up after baseline 20% or less? Were those lost to follow-up accounted for in the analysis?	NR	NR	✔	✔
10. Did the statistical methods examine changes in outcome measures from before to after the intervention? Were statistical tests done that provided *p* values for the pre-to-post changes?	✔	✔	✔	✔
11. Were outcome measures of interest taken multiple times before the intervention and multiple times after the intervention (i.e., did they use an interrupted time-series design)?	X	X	X	X
12. If the intervention was conducted at a group level (e.g., a whole hospital, a community, etc.) did the statistical analysis take into account the use of individual-level data to determine effects at the group level?	NA	NA	NA	NA

**Table 2:** Characteristics of included studies.

Study	Location	Study Type	Sample Size	Modality of Automated Discharge Instructions Used	Key Findings
Barga et al., 2025 [23]	United States	Exploratory cohort study	16	Personal virtual assistant (PVA) with tablet app	Adherence: ○ 78% for medications ○ 81% for exercise ○ 61% for surveys ○ 58% for specific tasks 80% patient endorsement
Chiu et al., 2022 [19]	United States	Quasi-experimental study	400	Automated discharge module with standardized instructions for elevated blood pressure	30-day follow-up for ED patients with elevated BP ○ 52.2% for pre-implementation ○ 48.4% for post-implementation ○ No significant difference No known hypertension—non-significant improvement in follow up Known hypertension—significant decrease in follow up
Chiu et al., 2021 [18]	United States	Quasi-experimental study	857	Automated program with tobacco cessation instructions	Significantly increased discharge instructions provided to tobacco users from 0.4% to 96%
Karasu et al., 2025 [34]	Turkey	Quasi-experimental study	57	SMS messages post-discharge after radical prostatectomy	Significantly improved quality of life in patients after radical prostatectomy
Leconte et al., 2019 [20]	France	Quasi-experimental study	7246	SMS messages post-discharge in an ambulatory surgery setting	Significantly higher patient contact rate than phone calls (86% vs. 57%), with early alert detection.
Lu et al., 2025 [26]	Taiwan	Descriptive correlational study	618	SMS-based discharge instructions from the ED	Improved patient knowledge, confidence, and satisfaction post-ED discharge
Ojeda et al., 2017 [27]	United States	Retrospective observational study	13,188	Automated call-back system for discharge from an urban tertiary hospital	592 patients reported 685 issues via post-discharge call-back line Approximately 25% of issues were already addressed in discharge instructions Resource barriers and medication questions were frequently unaddressed
Olsen et al., 2016 [24]	United States	Retrospective observational cohort study	6867	Automated phone assessments with hopes of reducing hospital readmissions	Readmission rates: 5.0% for live respondents 8.4% for non-respondents
Peuchot et al., 2020 [21]	France	Retrospective nonrandomized controlled study	4388	Pre- and post-op SMS reminders for surgical care	Conversion to full hospitalization 0.36% for SMS group 1.20% for the telephone call group The cost of SMS reminders was estimated to be half that of telephone calls
Rozanec et al., 2017 [13]	Canada	Prospective descriptive pilot study	22	Auto-generated discharge summary for radiotherapy	Automated summary improved communication and was valued by patients for continuity of care
Shuen et al., 2018 [31]	United States	Pilot feasibility randomized controlled trial	251	Automated phone call or SMS message 48 h post-discharge	Phone/SMS follow-ups Fewer ED revisits (>50% reduction) Fewer PMD/specialist contacts (approximate 30% reduction) Not statistically significant
Suffoletto et al., 2012 [32]	United States	Randomized controlled trial	144	Automated SMS message for antibiotic use and adherence	High engagement No significant improvement in adherence over printed instructions
Wright et al., 2018 [29]	United States	Quasi-experimental study	332	Automated phone calls post-discharge program for trauma patients	Automated calls to trauma patients identified follow-up needs 27% prompted nurse callbacks

**Table 3:** Summary of outcomes for readmission, ED revisit, and patient engagement across included studies. Criterion defined as NR: not reported; proxy-positive: indirect measure related to readmission; and suggestive: non-statistically significant trend in the favorable direction, based on preliminary or pilot data.

Study	Readmission Effect	ED Revisit Effect	Patient Engagement
Bargas-Ochoa et al., 2025 [23]	NR	NR	Positive
Chiu et al., 2022 [19]	Neutral	NR	Neutral
Chiu et al., 2021 [18]	NR	NR	Positive
Karasu et al., 2025 [34]	NR	NR	Positive
Leconte et al., 2019 [20]	NR	NR	Positive
Lu et al., 2025 [26]	NR	NR	Positive
Ojeda et al., 2017 [27]	NR	NR	Positive
Olsen et al., 2016 [24]	Positive	NR	Positive
Peuchot et al., 2020 [21]	Proxy-Positive	NR	Positive
Rozanec et al., 2017 [13]	NR	NR	Positive
Shuen et al., 2018 [31]	Neutral	Suggestive	Positive
Suffoletto et al., 2012 [32]	NR	NR	Positive
Wright et al., 2018 [29]	Suggestive	NR	Positive

## Data Availability

No new data were created or analyzed in this study. Data sharing is not applicable to this article.

## Supplementary Materials

The following supporting information can be downloaded at https://www.mdpi.com/article/10.3390/healthcare14060798/s1.
