# Supplementary material for: Automated Discharge Instructions in Medical and Surgical Care: A Systematic Review of Patient Engagement and Clinical Outcomes
Source: Healthcare (Basel). 2026 Mar 20;14(6):798. doi: 10.3390/healthcare14060798 (PMC13026212; doi:10.3390/healthcare14060798)
Supplement: Supplementary file 1 [file healthcare-14-00798-s001.zip › healthcare-4157804-supplementary.pdf]

Supplementary materials

1. Detailed search terms for each database search in this systematic review

Electronic Database	Search terms (including truncations)
PubMed; Embase; Web of Science; Scopus; CINAHL	Automated AND discharge AND instructions

2. Eligibility criteria for included articles in this systematic review

Inclusion Criteria	Exclusion Criteria
1. Study must be focused on the utilization of automated discharge instructions for patients post-hospital admission, emergency department visit or surgical stay.<br>2. Study must be peer-reviewed and original research.	1. Study is not focused on the utilization of automated discharge instructions for patients post-hospital admission, emergency department visit or surgical stay.<br>2. Study is not peer-reviewed or original research: correspondence, review articles, educational materials (book chapters), and non-peer review or retracted reports.<br>3. Study is not in English or has lack of English translation.
